# Supplementary material for: Continuous Glucose Monitoring (CGM) in Sports—A Comparison between a CGM Device and Lab-Based Glucose Analyser under Resting and Exercising Conditions in Athletes
Source: Int J Environ Res Public Health. 2023 Jul 25;20(15):6440. doi: 10.3390/ijerph20156440 (PMC10418731; doi:10.3390/ijerph20156440)
Supplement: Supplementary file 1 [file ijerph-20-06440-s001.zip › ijerph-2389456-supplementary.pdf]

Supplemental Information

	Δmean (mean$_{HC}$-mean$_{LC}$)	SD	T	df	95% CI$_{lower}$	95%CI$_{upper}$	Two-sided p-value	Effect size d
BL$_{CB}$	-3.29	11.27	-0.771	6	-13.71	7.15	.470	.39
BL$_{ISF}$	8.80	8.16	2.409	4	-1.34	18.94	.074	1.12
AUC$_{CB}$	-239.17	971.95	-.603	5	-1259.16	780.82	.573	.28
AUC$_{ISF}$	1714.00	732.07	4.683	3	549.11	2878.89	.018	1.62

Figure S1. Parametric test analysis of R/Fast comparing HC and LC, respectively considering BL and AUC.

Table S1. MARD ± SD of HC_R/Fast and LC_R/Fast

	MARD ± SD
HC_R/Fast	6.56 ± 5.56
LC_R/Fast	11.39 ± 6.25

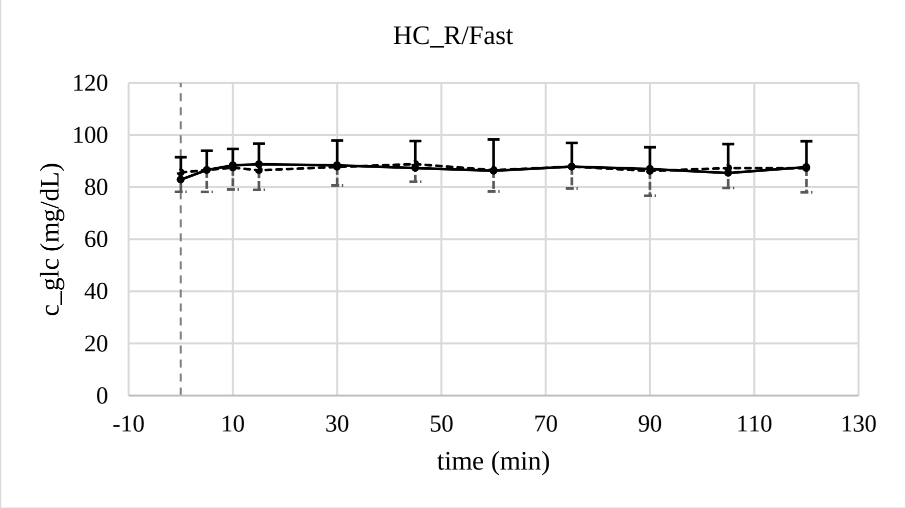

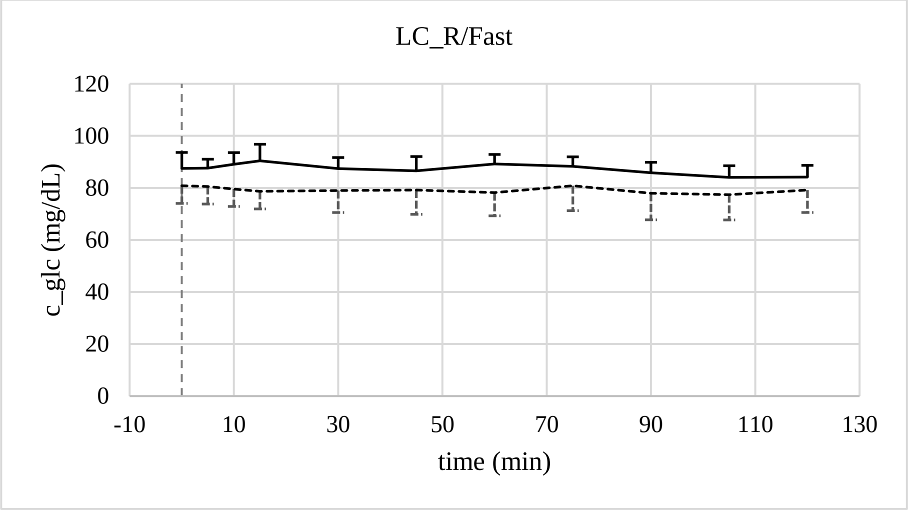

Figure S2. Mean CB and ISF glucose concentrations including SD (solid line and positive error indication reflecting SD = CB; dashed line and negative error indication reflecting SD = ISF).

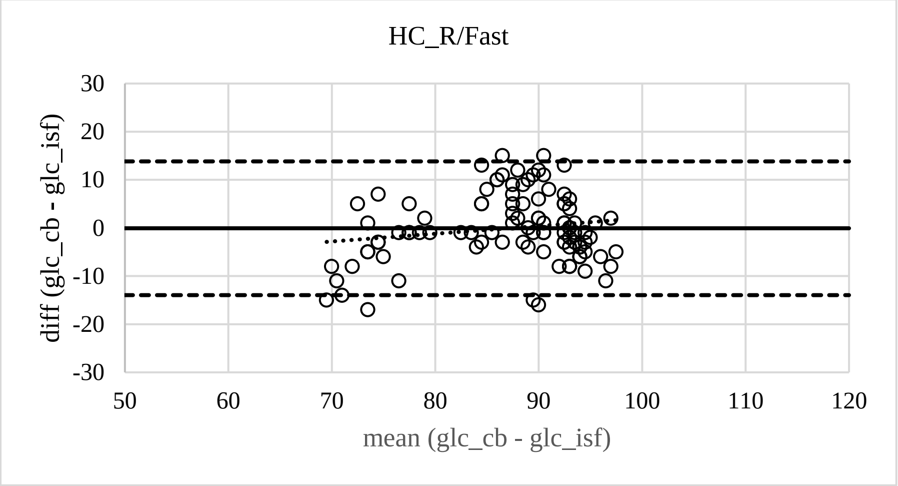

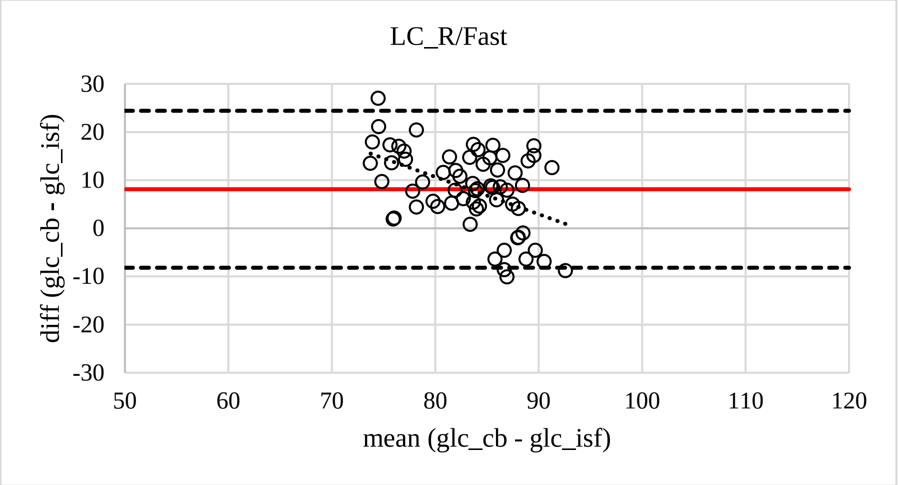

Figure S3. Systematic measurement difference of CB and ISF glucose concentration by Bland-Altman plot showing mean difference and their 95% confidence interval as lower and upper limit of agreement.

*Table S2. Parametric and non-parametric test analysis of R/Glc comparing HC and LC, respectively considering BL, PEAK and AUC. *As for AUC_{ISF} non-parametric test was performed, z-value instead of t-value and Pearson's r instead of Cohen's d are displayed.*

	Δmean ($mean_{HC}$-$mean_{LC}$)	SD	T*	df	95% CI_{lower}	95%CI_{upper}	Two-sided p-value	Effect size d*
BL_{CB}	-.60	7.69	-.247	9	-6.10	4.90	.811	.04
BL_{ISF}	.00	8.54	-.000	9	-6.11	6.11	1.000	.00
$PEAK_{CB}$	3.00	19.20	.469	8	-11.76	17.76	.652	.12
$PEAK_{ISF}$	4.14	15.89	.690	6	-10.55	18.84	.516	.23
AUC_{CB}	50.63	1042.89	.137	7	-821.25	922.50	.895	.05
AUC_{ISF}			-1.461				.144	.85

Table S3. Mean ± SD of CB and ISF glucose concentrations over time course of data sampling.

HC_R/Glc												
	BL	0	5	10	15	30	45	60	75	90	105	120

CB	87±5	92±11	113±21	130±20	146±22	177±24	166±22	143±22	114±30	99±27	79±17	74±14
ISF	78±12	76±13	82±14	94±17	115±23	157±21	158±22	125±25	94±22	80±21	72±13	68±16
LC_R/Glc												
	BL	0	5	10	15	30	45	60	75	90	105	120
CB	90±16	95±16	107±23	134±18	157±17	179±28	155±25	137±23	116±22	98±20	85±20	74±18
ISF	82±15	81±9	83±13	96±20	119±22	159±25	141±30	113±24	97±23	83±19	77±19	67±16

65/Glc											
	BL	0	10	20	30	40	50	60	70	80	90
CB	90±8	93±9	138±17	169±17	165±26	101±15	88±20	89±16	86±16	88±19	82±19
ISF	80±7	80±6	97±9	135±25	160±26	152±20	114±40	89±15	82±18	85±22	84±21
85/Glc											
	BL	0	10	20	30	40	50	60	70	80	90
CB	90±6	96±9	131±14	166±10	167±23	111±26	90±23	92±20	91±16	95±22	94±19
ISF	85±6	85±5	102±13	137±15	162±19	157±28	106±38	89±18	91±13	98±21	106±26

Table S4. MARD ± SD of R/Glc, MC_65/Glc and MC_85/Glc.

	MARD ± SD [%]
HC_R/Glc	16.73 ± 10.49
LC_R/Glc	16.88 ± 8.71
65/Glc	22.32 ± 24.10
85/Glc	18.19 ± 16.51
